# Autonomous Concrete Crack Monitoring Using a Mobile Robot with a 2-DoF Manipulator and Stereo Vision Sensors

**DOI:** 10.3390/s25196121

**Published:** 2025-10-03

**Authors:** Seola Yang, Daeik Jang, Jonghyeok Kim, Haemin Jeon

**Affiliations:** 1 Department of Civil and Environmental Engineering, Hanbat National University, Daejeon 34158, Republic of Korea; 20222114@edu.hanbat.ac.kr; 2 Department of Civil Engineering, The University of Texas at Arlington, Arlington, TX 76019, USA; jang.daeik@uta.edu; 3 Department of Mechanical Engineering, Gwangju Institute of Science and Technology, Gwangju 61005, Republic of Korea; enfycius@gm.gist.ac.kr

**Keywords:** concrete crack monitoring, autonomous mobile robot, 2-DoF manipulator, stereo vision, machine learning

## Abstract

Crack monitoring in concrete structures is essential to maintaining structural integrity. Therefore, this paper proposes a mobile ground robot equipped with a 2-DoF manipulator and stereo vision sensors for autonomous crack monitoring and mapping. To facilitate crack detection over large areas, a 2-DoF motorized manipulator providing linear and rotational motions, with a stereo vision sensor mounted on the end effector, was deployed. In combination with a manual rotation plate, this configuration enhances accessibility and expands the field of view for crack monitoring. Another stereo vision sensor, mounted at the front of the robot, was used to acquire point cloud data of the surrounding environment, enabling tasks such as SLAM (simultaneous localization and mapping), path planning and following, and obstacle avoidance. Cracks are detected and segmented using the deep learning algorithms YOLO (You Only Look Once) v6-s and SFNet (Semantic Flow Network), respectively. To enhance the performance of crack segmentation, synthetic image generation and preprocessing techniques, including cropping and scaling, were applied. The dimensions of cracks are calculated using point clouds filtered with the median absolute deviation method. To validate the performance of the proposed crack-monitoring and mapping method with the robot system, indoor experimental tests were performed. The experimental results confirmed that, in cases of divided imaging, the crack propagation direction was predicted, enabling robotic manipulation and division-point calculation. Subsequently, total crack length and width were calculated by combining reconstructed 3D point clouds from multiple frames, with a maximum relative error of 1%.

## 1. Introduction

Concrete structures are inherently susceptible to cracking owing to environmental factors, material degradation, and structural stress. Thus, timely maintenance interventions require early detection of defects and continuous monitoring of their structural integrity [1]. Traditional manual inspection methods are labor-intensive, time-consuming, and often subjective, thereby inadequate for consistent and reliable assessment. To address these limitations, advanced crack inspection methodologies have been proposed; these can be categorized into contact-based and non-contact-based techniques [2].

Contact-based methods include acoustic emission (AE) and ultrasonic testing. AE detects minute surface movements using high-frequency sound waves [3]; however, it requires specialized expertise, which limits its widespread adoption [4]. Although ultrasonic technology is effective for the detection of internal structures and defects [5,6], it struggles to identify micro-damage, especially in materials with low nonlinearity [7,8]. On the other hand, non-contact methods can be categorized into laser- and vision-based approaches. Despite the high precision of laser-based techniques, their high cost and complexity limit their widespread adoption. Consequently, vision-based structural health monitoring techniques have gained prominence, offering more cost-effective and accessible solutions, driven by advancements in computer vision and camera technology [9,10,11].

Image-processing-based crack-detection methods have evolved to address challenges such as irregular illumination, surface blemishes, and varying crack morphology. Various approaches have been developed, including subtraction and line-emphasis preprocessing [12], a modified iterated Hough transform to compensate for camera-position variations [13], and phase-symmetry-based crack-enhancement filters combined with thresholding and morphological algorithms [14]. In recent years, machine learning techniques have been increasingly incorporated into crack-detection methodologies. These approaches include restricted Boltzmann machines for crack identification in complex backgrounds [15], fully convolutional neural networks with encoder–decoder structures for pixel-wise classification [16], and adapted CNN models, such as AlexNet, for sliding-window detection [17]. For instance, a region-based convolutional neural network was employed to identify crack types and locations using minimal bounding boxes [1,18]. In addition to applying existing network architectures, studies have focused on modifying and optimizing networks for crack detection. Fu et al. enhanced crack-detection performance by modifying the DeepLabv3+ semantic segmentation algorithm, transforming the parallel structure of the atrous spatial pyramid pooling module into a densely connected form to improve multi-scale information encoding and expand the receptive field [19]. Li et al. proposed HrSegNet, a high-resolution network specifically designed for crack segmentation, which employs a dual-path architecture to preserve fine crack details and enhance contextual understanding. Its lightweight design and two-stage segmentation head ensure real-time inference [20].

Recent advancements in computational power and affordable robotic technologies have accelerated the development of unmanned aerial vehicles (UAVs) and unmanned ground vehicles (UGVs), which are now widely used in structural monitoring applications [21]. UAV systems have been demonstrated to be capable of autonomous inspection of buildings and quantification of cracks [22,23,24,25]. Yan et al. and Elamin and El-Rabbany proposed UAV-based crack-detection methodologies integrating high-resolution RGB imagery and LiDAR (light detection and ranging) data, utilizing deep learning techniques for automated concrete crack assessment and segmentation [22,23]. Ding et al. proposed a transformer-based crack detection and quantification UAV system, implementing a full-field-scale calibration method and an independent boundary-refinement transformer for pixel-level crack localization and measurement [24]. Jung et al. proposed an autonomous UAV-based bridge-inspection framework, integrating pre-flight 3D mapping with LiDAR and RTK-GPS (real-time kinematic global positioning system) and enabling structural surface inspection through autonomous navigation using sensor-fusion techniques in GPS-denied environments [25].

In another approach, Yu et al. proposed a UGV that maintains a constant distance from walls while capturing image data with a CCD (charge-coupled device) camera, combined with an image-processing-based system for crack detection and information extraction  [26]. Gibb et al. proposed a ground robotic inspection framework with multi-sensor integration, including ground-penetrating radar (GPR), electrical resistivity (ER) sensors, and camera systems, implementing fast and in-depth inspection modes and hybrid navigation techniques [27]. Yuan et al. proposed a vision-based crack-quantification method and projected the results onto 3D point clouds [28]. Ge and Sadhu mapped bridge damage using LiDAR-only KISS-ICP SLAM with an attention-enhanced YOLO (You Only Look Once) v7 network [29]. Yang et al. improved data efficiency with a semi-supervised YOLOv8 detector under limited labels [30]. Alkhedher et al. proposed a UAV–UGV cooperative system for pavement crack detection, improving coverage and efficiency [31]. While UAV-based monitoring platforms provide broad coverage, they are often limited by viewpoint instability, flight turbulence, and restricted depth accuracy, which can result in notable errors in crack quantification [30,31]. In addition, UAVs are less suitable for GPS-denied or confined indoor environments, where many structural inspections are required. Similarly, most UGV-based platforms lack flexible sensing capabilities, leading to reduced quantification accuracy and challenges in inspecting overhead or vertical surfaces. These limitations underscore the need for a system that integrates the high accuracy of localized inspection with the robustness of autonomous navigation and the flexibility to adapt to diverse inspection scenarios.

To overcome the aforementioned challenges, this study proposes a more flexible mobile robotic system equipped with a manipulator with 2 degrees of freedom (DoF) and machine learning capabilities as an innovative, automated crack detection and quantification framework. The system ensures comprehensive coverage without blind spots by combining autonomous navigation, advanced imaging techniques, and precise damage quantification. It utilizes simultaneous localization and mapping (SLAM) technology, enabling real-time tracking of the robot’s position and allowing it to follow predetermined paths on a pre-constructed map, thereby reducing the necessity of operator intervention and enhancing autonomy. When the machine learning framework detects any damage, the manipulator captures divided images of cracks. If the cracks are not contained within a single frame, they are subsequently stitched together using multiple images. Using a stereo vision sensor, the system also generates three-dimensional (3D) point cloud data based on binocular images, which are subsequently used for accurate crack quantification. The remainder of this paper is organized as follows. Section 2 details the robot and manipulator. Section 3 covers the data preparation and detection. Section 4 presents crack segmentation and quantification. Section 5 describes the experimental setup and results, and Section 6 concludes this paper.

## 2. Crack-Monitoring Robot with a 2-DoF Manipulator

In this study, a UGV equipped with stereo vision sensors was developed to detect cracks and generate a crack map. Stereo vision sensors were selected due to their cost-effectiveness and reliable depth accuracy at typical working distances, particularly when compared to the higher costs associated with LiDAR and the potential error sensitivity of structured-light systems, which can arise from issues related to laser manufacturing or installation positioning [32,33]. To facilitate crack detection over large areas, a 2-DoF manipulator with a stereo vision sensor mounted on its end effector was deployed. Another stereo vision sensor, mounted at the front of the robot, was used to acquire point cloud data of the surrounding environment, enabling tasks such as SLAM, path planning, tracking, and obstacle avoidance.

**Figure 1:**
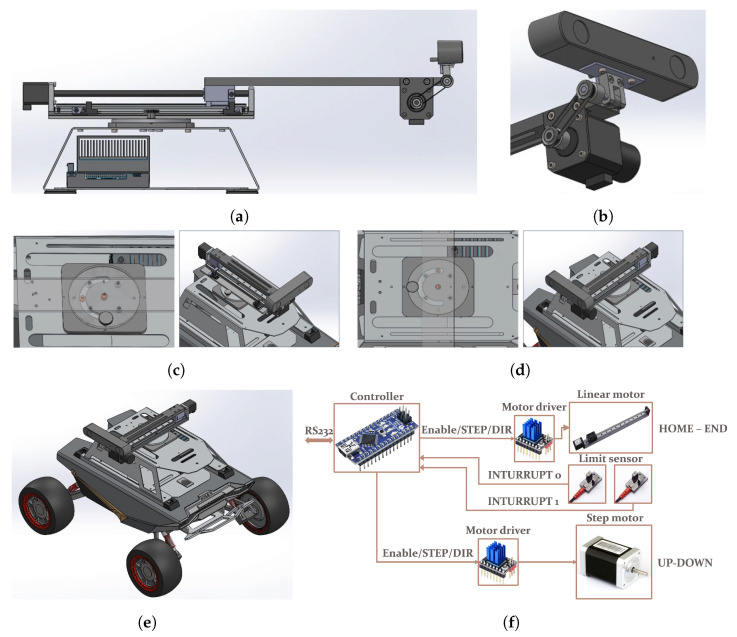
Conceptual design and system configuration of the 2-DoF motorized manipulator: (**a**) linear motion; (**b**) rotational motion; (**c**) front-facing installation of the vision sensor; (**d**) lateral-facing installation of the vision sensor; (**e**) UGV model equipped with the manipulator; (**f**) electrical system configuration.

**Figure 2:**
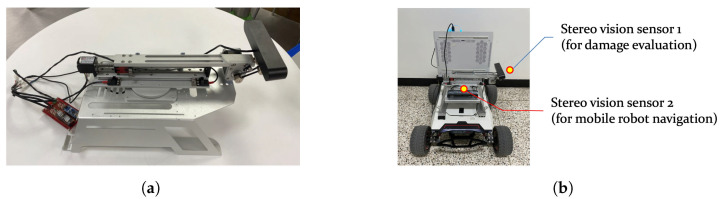
UGV and manipulator configuration: (**a**) manipulator module; (**b**) UGV front view with the manipulator installed.

To monitor structural conditions, a 2-DoF manipulator capable of linear and rotational movements was designed and manufactured. The motorized manipulator, capable of multi-directional rotation, was mounted on top of the mobile robot, as shown in Figure 1. In this figure, the proposed 2-DoF motorized manipulator is integrated with a manual rotation plate, enabling versatile adjustment of sensor orientation toward the lateral and forward directions. The linear motor primarily functions to stabilize the rotational trajectory during directional reconfiguration while also facilitating close-range inspection of cracks when necessary. In conjunction with rotational actuation, this design ensures a consistent and reliable rotational path of the camera module, thereby enhancing observational stability and spatial coverage relative to conventional single-DoF configurations. The manipulator provides a rotation-angle range of 165° and a linear stroke length of 200 mm, with motion precisions of 0.05 mm for the linear module and 1.8° for the rotational module. One-to-one communication between the controller and computer was established via RS-232 with a baud rate of 115,200, 8 data bits, no parity, one stop bit, and no flow control. The linear and stepping motors were then driven and controlled using a motor driver. A message protocol comprising four data phrases (header, address, command, and checksum) was constructed between the computer and the controller and was used to control the forward movement, backward movement, and movement termination of the linear motor and the upward rotation, downward rotation, and rotation termination of the step motor. The stereo vision sensor mounted on the manipulator’s end effector was utilized for crack detection and quantification, as well as manipulator motion control, whereas the front-mounted stereo vision sensor was utilized for the robot’s mobility, as illustrated in Figure 2.

In this study, an adaptive resampling-based grid-mapping algorithm utilizing the Rao–Blackwellized particle filter (RBPF) was applied to address the SLAM problem [34]. The RBPF approach is known to be an effective method for SLAM, where each particle represents an individual environmental map. By introducing adaptive techniques for particle reduction, including an accurate proposal distribution that considers robot movement and recent observations, and by employing selective resampling strategies, this approach significantly reduces pose uncertainty and mitigates particle depletion issues. Considering the confined, small-scale, and static nature of the indoor environment, grid-based SLAM was selected for its computational simplicity and efficiency [35]. While graph-based SLAM provides superior accuracy and scalability in larger, dynamic, and complex environments, the grid-based approach offers sufficient precision and faster processing, making it suitable for the experiments conducted in this study [36]. The autonomous navigation stack of the mobile robot, including SLAM, obstacle avoidance, and path-following algorithms, was implemented using the Robot Operating System (ROS) Melodic Morenia framework on the Ubuntu 18.04 (Bionic) operating system.

The crack information obtained from the manipulator-mounted sensor was superimposed onto the generated map after considering the relative positions of the two stereo vision sensors and the length of the manipulator, based on a coordinate transformation relationship using Equation (1). In the equation, *x* and *y* represent the lateral and longitudinal distance differences between the two sensors with respect to the frame of the robot, ψ denotes the rotation angle of the manipulator, and $C_{\theta }$ and $S_{\theta }$ represent cosθ and sinθ, respectively.

(1) Ts2s1=100x010y001z000110000Cθ−Sθ00SθCθ00001Cϕ0Sϕ00100−Sϕ0Cϕ00001Cψ−Sψ00SψCψ0000100001

## 3. Crack Data Preparation and Detection

**Figure 3:**
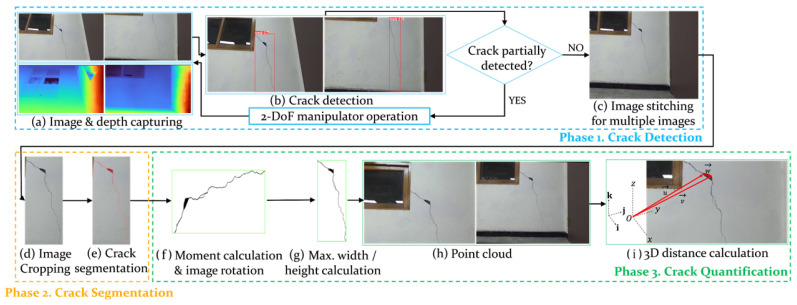
Flowchart for manipulator operation and crack quantification.

This study developed a three-stage methodology for crack analysis in structural environments, as depicted in the flowchart in Figure 3. The process encompassed deep learning-based crack detection coupled with strategic image cropping, followed by high-precision deep learning-based crack segmentation and quantification based on point clouds generated from images. In this approach, coarse bounding-box information was utilized to enhance segmentation precision within the cropped regions, leading to more accurate pixel-level crack identification [37,38,39]. The initial phase employed an advanced deep learning algorithm for real-time crack detection, enabling mobile robots to conduct efficient, autonomous scans of structural surfaces. This system dynamically assesses the presence and extent of cracks and determines the necessity of manipulator activation and subsequent image acquisition. Upon crack detection, the system executes image stitching to create a comprehensive visual representation of the inspected area. In the second phase, the stitched images underwent a refined cropping process to isolate the regions of interest containing the initially detected cracks. The cropped sections were then subjected to a state-of-the-art segmentation algorithm, facilitating pixel-level crack delineation. This approach significantly enhanced the accuracy and level of detail of crack identification. The final stage of the process leveraged stereo vision technology to acquire depth images and point cloud data. This three-dimensional information is crucial for the precise quantification of the segmented cracks, yielding accurate measurements of crack dimensions and characteristics. The contents of the first phase are discussed in Section 3, and the details of the second and third phases are presented in Section 4.

### 3.1. Dataset for Crack Analysis

**Figure 4:**
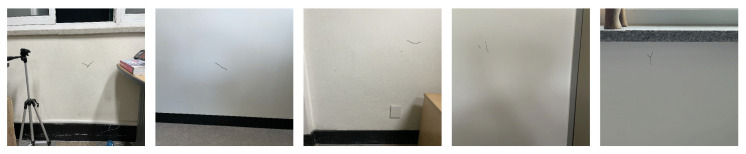
Image synthesis results for indoor crack simulation.

**Figure 5:**
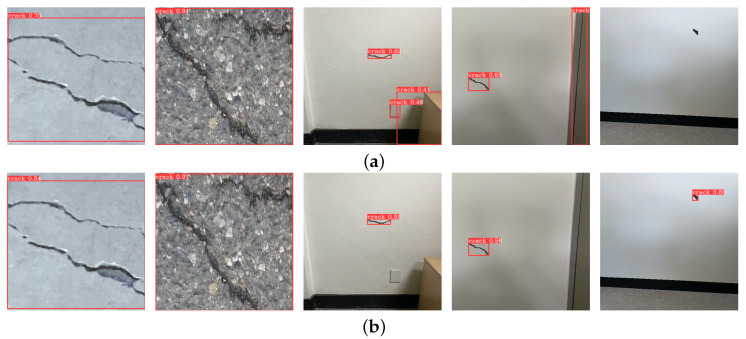
Crack-detection results: (**a**) without and (**b**) with synthetic images.

A dataset for crack detection was developed by leveraging two pre-existing concrete crack image datasets, the Concrete3k dataset [40] and the CrackSeg9k dataset [41], as primary training data, supplemented with newly synthesized crack images to enhance detection performance in indoor structural environments. While these existing datasets predominantly contain full-frame crack images, they often fail to accurately capture small or thin cracks on wall surfaces, which are critical for indoor structural inspection. To address this limitation and replicate crack scenarios accurately within interior structural settings, an image-synthesis technique was employed by combining fine crack patterns with diverse indoor spatial backgrounds. Owing to the operational limitations of the stereo vision sensor, which required maintaining a predetermined distance from the crack, the acquired images inherently captured cracks, including the surrounding background. This necessitated the synthesis of crack images embedded within broader wall backgrounds, as opposed to using isolated, full-frame crack images, as shown in Figure 4. Synthetic crack images were generated in Python (version 3.9.16) using the OpenCV library by cropping crack regions from segmentation-labeled images, applying random scaling and in-plane rotation, and compositing them via alpha blending onto five indoor white-wall background images. White-wall candidate regions were detected using HSV near-white thresholding and morphological filtering; rectangular regions of interest were then verified and used to randomly place the scaled cracks while avoiding boundary overlap and inter-crack collisions. This approach not only enriched the diversity of the dataset but also enhanced its relevance to indoor structural inspection scenarios. By integrating established datasets with custom-synthesized images, a robust foundation was created for training and evaluating crack-detection algorithms in various indoor structural contexts. The performance evaluation, including the synthesized images, is presented in Table 1 and Figure 5. The table presents two mean average precision (mAP) metrics for evaluating detection performance. The first metric is calculated using a fixed intersection-over-union (IoU) threshold of 0.5, representing the average precision across various recall levels for each class. The second metric provides a more comprehensive assessment by averaging mAP values computed at multiple IoU thresholds, ranging from 0.5 to 0.95 in increments of 0.05. This broader evaluation captures detection performance across varying degrees of overlap between predicted and ground-truth bounding boxes. As shown in Table 1 and Figure 5, the performance improvement can be attributed to the inclusion of synthetic images that explicitly compensate for the under-representation of thin and small cracks in existing datasets.

**Figure 6:**
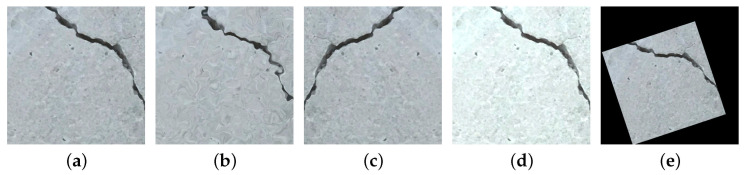
Augmentation of the training dataset: (**a**) original image, (**b**) elastic transformation, (**c**) horizontal flip, (**d**) color jittering, (**e**) affine transformation.

All images underwent preprocessing steps, including resizing to a uniform resolution of 500×500 pixels and normalization. Subsequently, data augmentation techniques were employed to enhance the diversity of the training dataset. Four distinct augmentation methods, namely elastic transformation, horizontal flipping, color jittering, and affine transformations, were applied, as illustrated in Figure 6. Through this process, the original dataset of 16,965 images, including synthetic images, was expanded to approximately 84,825 images. The augmented dataset was then partitioned into three subsets: 60% for training, 20% for validation, and the remaining 20% for testing purposes.

### 3.2. Real-Time Deep Learning Network for Crack Detection

The YOLOv6-s object-detection algorithm was applied to enable real-time concrete crack detection on mobile robots. YOLOv6 is a single-stage object-detection framework that uses a single pass through the network to perform both object detection and classification [42]. It employs an efficient re-parameterizable backbone called EfficientRep. The main component of the backbone is the RepBlock, which is transformed into stacks of 3×3 convolutional layers with ReLU activation functions (called RepConv) during inference. In this study, YOLOv6-s, a relatively lightweight variant of YOLOv6 that offers a good balance between performance and efficiency, was adopted. For the YOLOv6-s model, stochastic gradient descent (SGD) was utilized as the optimizer, while the cosine decay technique was employed as the learning rate scheduler, following the approach described in [42]. The following hyperparameters were used for YOLOv6-s: an initial learning rate of 0.01 with a cosine schedule ending at 0.01, a momentum of 0.937, and a weight decay of $5\times 10^{-4}$. A three-epoch warm-up was applied with a warm-up momentum of 0.8 and a bias learning rate of 0.1. Batch normalization used eps $1\times 10^{-3}$ and a momentum of 0.03. For data augmentation, adjustments to the HSV (Hue, Saturation, Value) channels were randomly applied, and random geometric transformations, including rotation, scaling, and translation, were implemented. As a result, the YOLOv6-s model achieved an F1 score of 0.95, a precision of 0.95, a recall of 0.94, and processed images at approximately 241 FPS. The experiments were conducted on a workstation equipped with dual NVIDIA RTX 3080 Ti GPUs (NVIDIA Corporation, Santa Clara, CA, USA), an AMD Ryzen 5900x CPU (Advanced Micro Devices, Inc., Santa Clara, CA, USA), and 48 GB of RAM. For this evaluation, the detection performance threshold was set to 0.5, and the frames per second (FPS) were calculated by dividing 1000 milliseconds by the total time required per frame, including preprocessing, inference, and non-maximum suppression.

### 3.3. Crack Stitching with the 2-DoF Manipulator

In cases where cracks are detected using the YOLOv6-s model, or when cracks are only partially detected, a 2-DoF manipulator is activated to scan the surrounding area. Image-stitching techniques are then utilized to integrate the segments and analyze the crack’s size. Within each captured image, distinctive invariant features are detected. These features are invariant to image scale and rotation, providing robust matching across a substantial range of affine distortions, 3D viewpoint changes, noise, and illumination variations [43].

To match the keypoint features extracted from the segmented images, a brute-force descriptor matcher is employed. This matcher compares the descriptor of each feature in an image against all descriptors in other images, performing the matching operation. For each feature, a *k*-nearest neighbor search with *k* = 2 was performed, and the distances of the closest and second-closest neighbors were compared to determine match uniqueness [44,45]. The matching candidates are then evaluated by calculating the Euclidean distance, and those within a specific threshold are identified as matching points [46]. After extracting the coordinates of the identified matching points, their homography is calculated using the MAGSAC (Marginalizing Sample Consensus)++ algorithm [47], which improves robustness through iteratively re-weighted least-squares fitting, providing higher accuracy and reliability compared to other homography estimation algorithms, such as RANSAC (random sample consensus), graph-cut (GC) RANSAC, and MAGSAC, under challenging conditions [48,49]. The homography matrix represents a projective transformation, being a non-singular 3×3 matrix with 8-DoF. The relationship between matching points in two images and the homography matrix is expressed in Equation (2), where *x* and $x^{\prime }$ denote the matching points in the first and second images, respectively [50].

(2) $$\begin{array}{c} x^{\prime }=Hx \end{array}$$

By transforming one image onto the coordinate system of another image using the computed homography, a stitched image is obtained. The homography matrix was estimated using MAGSAC++ with a second nearest-neighbor threshold of 0.85, a noise standard deviation threshold of σ=1.0 pixels, a probabilistic weighted sampler of 4, and iteration and confidence settings of 10,000 and 0.99, respectively.

## 4. Crack Segmentation and Quantification

### 4.1. Application of Deep Learning Networks for Crack Segmentation

To achieve high-precision, pixel-level detection of structural surface cracks, state-of-the-art segmentation networks, including SFNet, PP-MobileSeg, RTFormer, and HrSegNet, were applied. SFNet introduces the Flow Alignment Module (FAM), which learns semantic flow between adjacent feature-map levels and efficiently propagates high-level features to high-resolution features [51]. SFNet with ResNet (Residual Network) -18 was chosen for crack segmentation because its Flow Alignment Module (FAM) is particularly effective for thin, boundary-dependent structures such as cracks. FAM enriches feature maps with semantic and spatial information by learning semantic flow between adjacent feature maps, enabling accurate transfer of deep semantic information to high-resolution layers. This improves boundary recovery and enhances the representation of narrow crack patterns while maintaining computational efficiency and suitability for real-time crack mapping.

**Figure 7:**
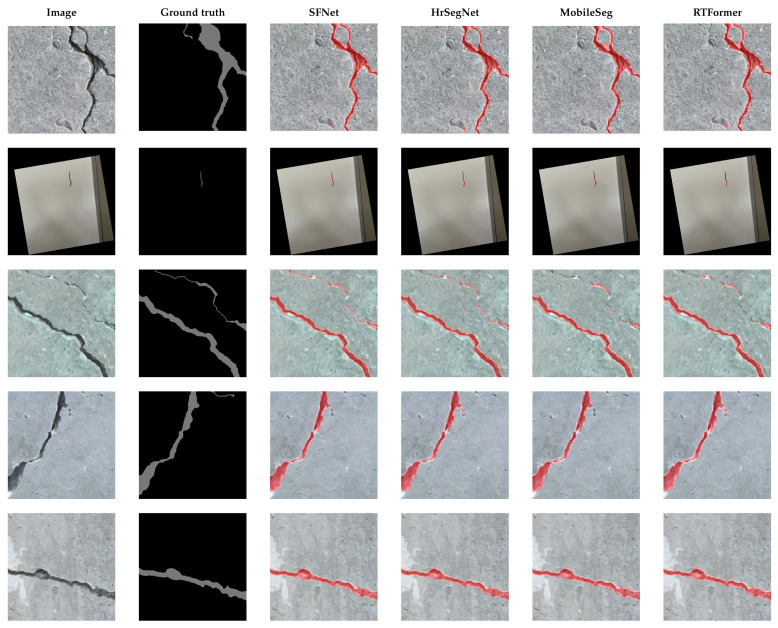
Segmentation results using SFNet (ResNet-18), HrSegNet, MobileSeg (MobileNetV3-Large), and RTFormer.

**Figure 8:**
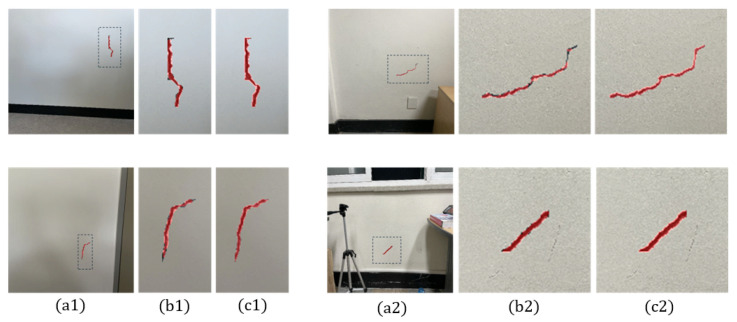
Comparison of segmentation results: (**a1**, **a2**) original captured image; segmentation without (**b1**, **b2**) and with (**c1**, **c2**) image pre-segmentation processing, such as deep learning-based region-of-interest detection, cropping, and scaling.

In this paper, SFNet was selected as the precise and real-time segmentation network, and the segmentation performance based on single-run training was compared with that of other state-of-the-art architectures such as HrSegNet, MobileSeg, and RTFormer, as shown in Table 2 and Figure 7. Although the segmentation performance across these models is relatively close in terms of mIoU, the trade-off between accuracy and computational efficiency is critical in robotic applications. SFNet (ResNet-18) achieves competitive accuracy (mIoU = 81.3%) with lightweight computation (13.8M parameters), thereby ensuring real-time inference on embedded robotic platforms. To evaluate the effectiveness of SFNet, a dataset specifically designed to represent cracks occupying a relatively small proportion of wall surface images was utilized. The segmentation performance is enhanced by employing object detection followed by image cropping and scaling, rather than performing segmentation directly on the captured images. This approach addresses the challenge of segmenting objects that occupy a small proportion of the overall image. By increasing the relative size of the object of interest through cropping and scaling within the deep learning-based object-detection framework, the accuracy of subsequent segmentation tasks is improved. Figure 8 illustrates a performance comparison between two crack-segmentation approaches: (1) utilizing direct segmentation on the original image, and (2) employing a sequential process of object detection, image cropping, and scaling prior to segmentation. The latter method resulted in the crack occupying a larger proportion of the input image for the segmentation task, with enhanced visibility of fine details. A dataset of 1187 such images was utilized for the experiments. The baseline segmentation results, obtained without any post-processing, yielded a mean IoU (mIoU) of 75.9%. In contrast, applying the proposed post-processing techniques, such as cropping and scaling, significantly improved segmentation performance, yielding an mIoU of 82.1%.

### 4.2. Crack Quantification Using Contour-Moment Computation

The crack-segmentation results are analyzed to calculate crack dimensions based on the corresponding point cloud data. By using reconstructed three-dimensional point clouds, the image distortion caused by non-vertical capturing can be corrected. When the crack size exceeds the field of view and is captured across multiple divided images with different view angles, only the matched features within the bounding box of the crack are filtered using the MAGSAC++ algorithm. Subsequently, the distances between the bounding-box edge in the crack’s progression direction and the filtered features are calculated, and the point closest to the edge is selected as the division point. Using this reference point, the distances between the point clouds matched to each divided image are computed and summed to determine the total crack length. The pseudo-code for measuring the total crack length based on stitched images can be found in Algorithm 1.

To robustly measure the width and length of cracks regardless of their orientation, the moments of the contour of the segmented crack were computed. The process involves meticulously extracting the crack’s contour, determining the principal axis along its length, and accurately calculating the width perpendicular to the axis. The methodology employs the following equation to compute the moments of each contour pixel:

(3) $$\begin{array}{c} M_{ij}=\sum_{x}\sum_{y}x^{i}y^{j}I\left(x,y\right) \end{array}$$

From Equation (3), the center point of the contour is derived using the following equation:

(4) $$\begin{array}{ccc} c_{x} & =\frac{M_{10}}{M_{00}}\;c_{y} & =\frac{M_{01}}{M_{00}}\; \end{array}$$

where $M_{00}$, $M_{10}$, and $M_{01}$ are defined as follows:

(5) $$\begin{array}{cccc} M_{00} & =\sum_{x}\sum_{y}I(x,y),\;M_{10} & =\sum_{x}\sum_{y}x\cdot I(x,y),\;M_{01} & =\sum_{x}\sum_{y}y\cdot I(x,y). \end{array}$$

In Equation (5), I(x,y) represents the pixel value at coordinates (x,y) in the image. $M_{00}$ is the zeroth-order moment, indicating the total brightness or sum of pixel values, which is related to the image area. $M_{10}$ and $M_{01}$ are the first-order moments for the *x* and *y* axes, respectively, used to calculate the center of mass in each direction. The rotation angle is determined based on the shape of the bounding box relative to the contour’s center. The principal axis is defined as the longer direction of the detected bounding box from the contour endpoints; the mask is then aligned with this axis, and the crack width is measured orthogonal to it as the maximum cross-sectional span. For branched cracks, the width is measured along cross-sectional profiles orthogonal to the principal axis. At each profile, all contiguous intersection intervals with the crack mask are identified, and the longest interval is retained. The global crack width is then defined as the maximum length among these retained intervals across all profiles.
**Algorithm 1** Crack-stitching algorithm using MAGSAC++1:**Variables:**2:$e_{f}$, $e_{n}$: farthest and nearest points among segmented pixels based on the manipulator’s motion3:**Input:** Set of segmented images and corresponding point clouds4:**Output:** 3D point cloud-based stitched crack length5:**for** i=1 to $N_{s}-1$ **do**             % $N_{s}$: number of segmented frames6:    Detect bounding box $B_{c}$ enclosing segmented regions7:    Calculate $P_{i}$ and $P_{i+1}$ using MAGSAC++    %$P_{i}$: matching points in *i*-th frame8:    Filter $P_{i}^{\prime }$ and $P_{i+1}^{\prime }$ within $B_{c}$9:    **if** i=1 **then**10:        $p_{t,i}\leftarrow \arg\max_{p\in P_{i}^{\prime }}d\left(p,e_{f}\right)$           % d(m,n): distance between m,n11:        **for** j=1 to $N_{t}$ **do**                    % $N_{t}$: total frames12:           $l_{i}\leftarrow l_{i,j}\cup d\left(p_{t,i},e_{f}\right)$13:        **end for**14:    **else**15:        $p_{s,i}\leftarrow \mathrm{match}\left(p_{t,i-1}\right)$              % match($p_{t,i-1}$): matched cloud16:        $p_{t,i}\leftarrow \arg\min_{p\in P_{i}^{\prime }}d\left(p,e_{n}\right)$17:        **for** j=1 to $N_{t}$ **do**18:           $l_{i}\leftarrow l_{i,j}\cup d\left(p_{s,i},p_{t,i}\right)$19:        **end for**20:    **end if**21:    $l_{t}\leftarrow l_{t}+l_{i}$                 % $l_{t}$: total length, $l_{i}$: length in frame *i*22:**end for**23:$p_{s,N_{s}}\leftarrow \mathrm{match}\left(p_{t,N_{s}-1}\right)$24:**for** j=1 to $N_{t}$ **do**25:    $l_{N_{s}}\leftarrow l_{N_{s},j}\cup d\left(p_{s,N_{s}},e_{n}\right)$26:**end for**27:$l_{t}\leftarrow l_{t}+l_{N_{s}}$

## 5. Experiments

### 5.1. Experimental Setup

To validate the performance of the proposed autonomous crack-monitoring system, which integrates a UGV equipped with a manipulator and stereo vision sensors, indoor experimental tests were conducted. As a preliminary step, the performance of the stereo vision sensor was thoroughly evaluated to ensure its reliability for the subsequent experiments. In the experiments, the ZED 2i stereo vision sensor by Stereolabs, with a fixed baseline length of 120 mm, was used in Neural Plus mode with the latest SDK version (4.1.2). The intrinsic and extrinsic parameters were verified using a checkerboard-based calibration procedure, yielding a mean reprojection error of 0.17 ± 0.02 pixels across both lenses, confirming reliable stereo geometry. The stereo camera, configured at 2K resolution (2208×1242 pixels), achieved a spatial resolution of approximately 1 cm at close range, making it suitable for the current application of crack mapping.

**Figure 9:**
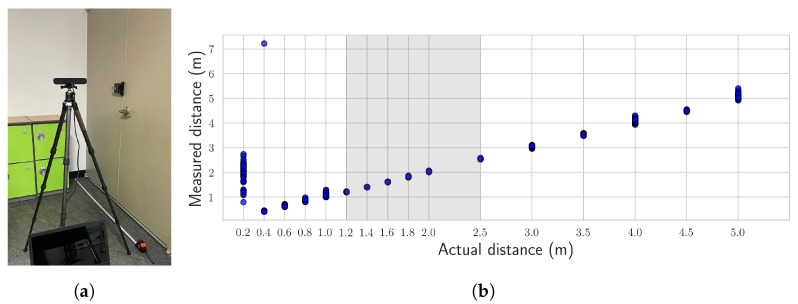
Validation of depth measurement for the stereo vision sensor: (**a**) experimental setup, (**b**) results.

**Figure 10:**
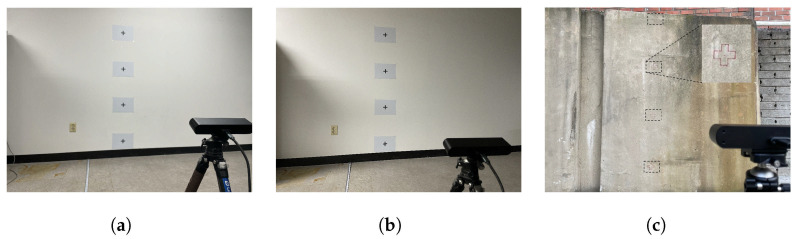
Experimental setup under different conditions: (**a**) normal lighting with a smooth surface, (**b**) low lighting with a smooth surface, and (**c**) normal lighting with a concrete surface.

To evaluate the performance of the stereo vision sensor, two sets of experiments were conducted: (1) analyzing the quality of point clouds generated at various distances, as shown in Figure 9, and (2) measuring horizontal and vertical dimensions under varying lighting conditions, surface types, and view angles, as shown in Figure 10. The point cloud data were collected 100 times at each distance, ranging from 0.2 m to 5 m. The results confirmed stable point cloud generation within the 1.2 to 2.5 m range. Based on these findings, four markers were installed on a wall at a distance of 2 m from the sensor, and their dimensions were measured. The markers were positioned at four relative heights with respect to the sensor: at the same height, 50 cm below, and 50 cm and 100 cm above. To enhance the robustness of the measurements, outliers observed during the experiments were addressed using robust statistical methods. The median absolute deviation (MAD) was employed to remove outliers, using a linear 1D kernel with K=15 and outlier detection thresholds of 3.5 within each frame and 3 across frames (see Table 3). After outlier removal, the results demonstrated stable measurements with relative errors within 1%, regardless of lighting conditions, wall-surface types, or view angles. The distortion caused by non-vertical image capturing was corrected through the reconstruction of the filtered 3D point clouds [54].

**Figure 11:**
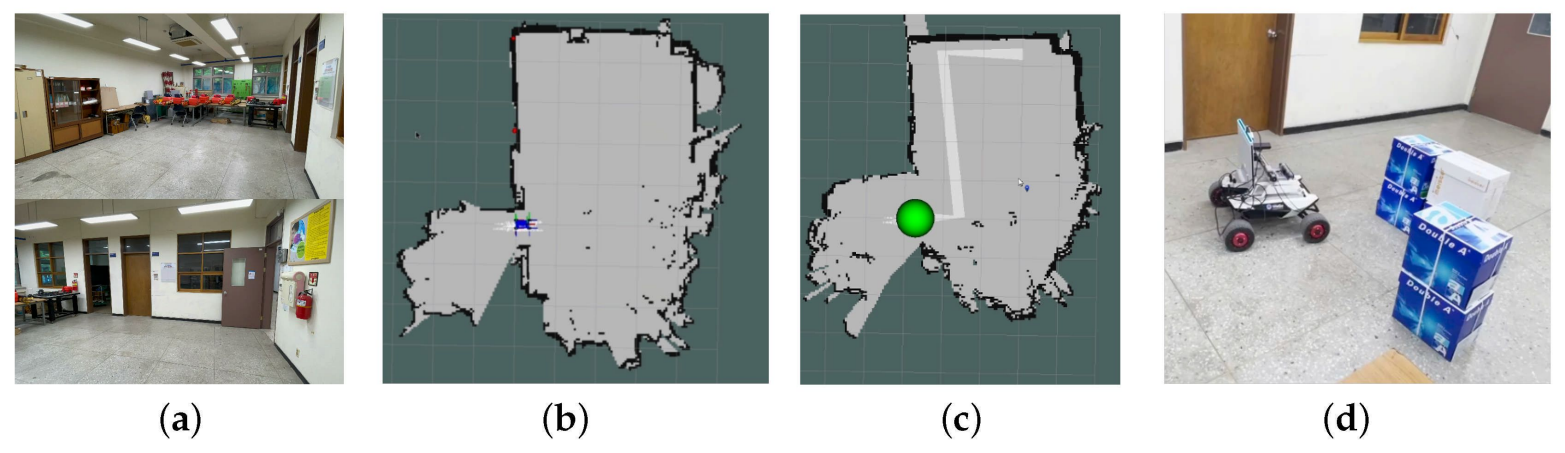
Key technologies for autonomous navigation and map building: (**a**) indoor experimental environment, (**b**) SLAM, (**c**) path generation and following, (**d**) obstacle avoidance.

The test environment featured concrete walls with a horizontal and a vertical crack, simulating typical structural defects (see Figure 11a). The experimental cracks, observed on a reinforced concrete wall of a Korean building constructed in 1997, are considered representative, in-service defects likely associated with long-term differential settlement and shrinkage [55,56]. For autonomous real-time monitoring of these concrete cracks, the Scout Mini robot manufactured by WeGo (Yongin, Republic of Korea) was selected due to its high stability in various environments. The Scout Mini measures 612 × 580 × 245 mm and weighs 23 kg, featuring a maximum payload capacity of 10 kg with its standard wheels. The manipulator mounted on the UGV operated has a rotational speed of approximately 4.7 RPM and a linear translational speed of about 45 mm/s, values that were verified through motor driver settings and pulse-based calculations. The autonomous navigation capabilities of the robot, including map generation, path planning, and obstacle avoidance, are illustrated in Figure 11b–d, respectively. The main parameters used for the autonomous navigation of the mobile robot are summarized in Table 4. In particular, the map grid cell size for the SLAM process was set to 5 cm, which was selected as a balance between mapping precision and computational efficiency, considering the scale of the experimental environment and the target cracks.

For the deep learning techniques used in crack detection and segmentation, a workstation was configured with an AMD Ryzen 9 5900X 12-Core Processor running at 3.70 GHz, 32 GB of RAM, and dual NVIDIA GeForce RTX 3080 Ti graphics cards in a parallel GPU configuration. The deep learning models were trained with the following hyperparameters. For the detection model, the number of epochs was set to 100, with a batch size of 6 per GPU. SGD was used as the optimizer, and a cosine scheduler was applied. The varifocal loss was employed to calculate classification loss, and the bounding-box loss was used to measure the similarity between predicted and ground-truth bounding boxes. In the segmentation model, the batch size per GPU was set to 2, allowing for efficient gradient updates and optimal GPU utilization. SGD was also employed as the optimizer, with online hard example mining cross-entropy loss used as the loss function. The initial learning rate was set to 0.01, and a polynomial decay scheduler was implemented. For real-time inference and SLAM implementation on the mobile robot platform, the trained model was deployed to a portable system featuring an NVIDIA RTX 3080 Ti laptop GPU, an Intel i7-12700H processor, and 32 GB of RAM. Experimental validation was conducted to verify the real-time operational capability of the model deployed on the mobile robot platform.

### 5.2. Experimental Results

**Figure 12:**
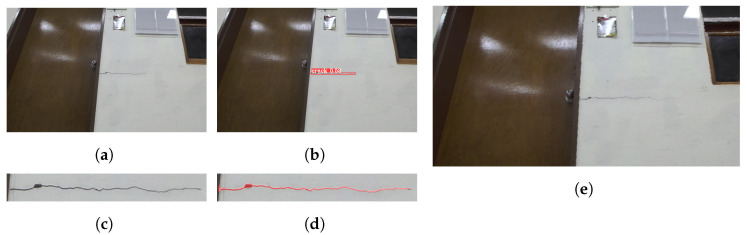
Quantification process of the first crack: (**a**) captured crack image, (**b**) crack detection result, (**c**) extracted detected area, (**d**) segmentation result, (**e**) point cloud visualization.

**Figure 13:**
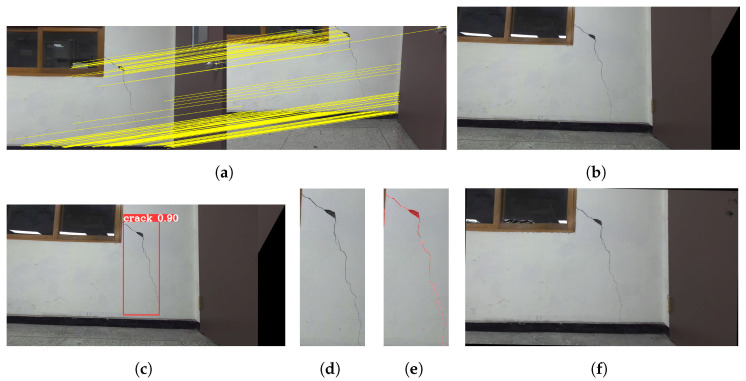
Quantification process of the second crack: (**a**) matched features, (**b**) stitched crack image, (**c**) crack-detection result, (**d**) extracted detected area, (**e**) segmentation result, (**f**) point cloud visualization.

**Figure 14:**
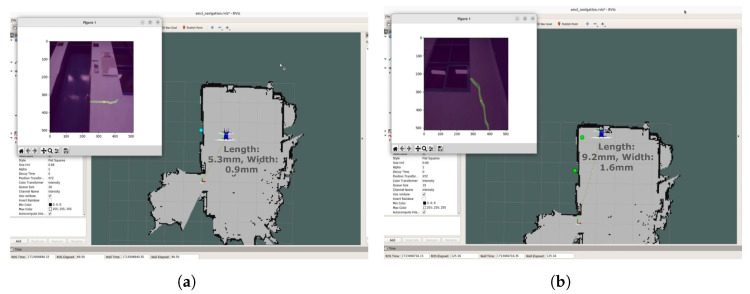
Real-time crack mapping from two separate drives: (**a**) first crack captured during the first drive, (**b**) second crack captured during the second drive.

In the experiments, the robot navigated to build a map of its surroundings, detecting and quantifying cracks along user-specified paths. It dynamically avoided obstacles while marking detected cracks on a constructed map for users to access detailed crack information by location, ensuring a localization accuracy of less than 20 cm. In the indoor experimental environment, there were horizontal and vertical cracks in separate regions. The robot first captured images of the horizontal crack, determined whether manipulator movement was required after crack detection, and produced the cropping and segmentation results shown in Figure 12. In the experiments with the first horizontal crack, the mAP and IoU values of crack detection and segmentation were 89.6% and 71.9%, respectively. The second crack extended beyond the frame, necessitating the use of the 2-DoF manipulator to move along the crack’s direction, allowing for the stitching of multiple images to obtain a complete view. The process of crack quantification, including image stitching based on manipulator movements, is illustrated in Figure 13. In the experiments with the second vertical crack, the mAP and IoU values of crack detection and segmentation were 79.6% and 71.2%, respectively. Figure 14 shows the map created in real time based on SLAM, with the crack locations sequentially marked. After two runs, it was confirmed that the crack locations were consistently marked in the same positions. The quantified crack sizes were compared as described below.

**Figure 15:**
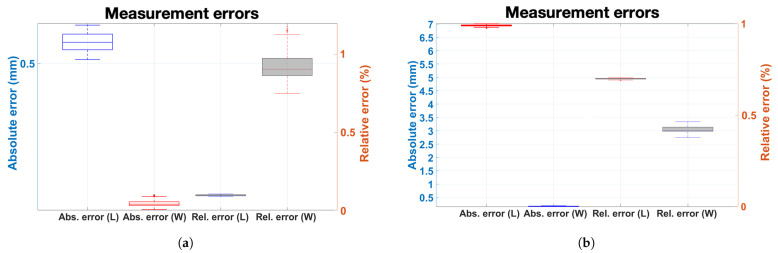
Box plots of absolute and relative errors in measured cracks with the median absolute deviation: (**a**) first crack, (**b**) second crack.

The first crack measured 588 mm in maximum length and 10 mm in maximum width, while the second crack exhibited larger dimensions of 995 mm in length and 39.5 mm in width. Measurement accuracy was assessed through absolute and relative error analyses, with crack length and width compared using a tape measure with a resolution of 1 mm and a crack gauge ruler with a resolution of 0.05 mm, respectively. For the first crack, width measurements yielded a maximum absolute error of 0.10 mm and a maximum relative error of 1.00%. The length measurement showed a maximum absolute error of 1 mm and a maximum relative error of 0.17%. Despite its larger size, the second crack demonstrated comparable measurement precision. The width measurement showed a maximum absolute error of 0.15 mm and a maximum relative error of 0.38%. The length measurement exhibited a maximum absolute error of 7 mm and a maximum relative error of 0.70%. Both cracks were measured 100 times, and the results of these repeated measurements are summarized in Table 5 and Figure 15. The average end-to-end processing time, including image acquisition, crack detection, manipulator path planning, and mapping, was measured as 6.46 s per crack on the laptop GPU (RTX 3080 Ti, NVIDIA Corporation, Santa Clara, CA, USA), confirming the feasibility of the proposed system for real-time crack monitoring.

Based on previous studies, each state-of-the-art robotic crack-monitoring approach has inherent limitations. UAV-based systems are effective for large-area coverage but often exhibit quantification errors due to viewpoint instability, flight turbulence, and limited depth resolution [57,58]. In contrast, the proposed system achieved relative errors at or below 1% for both crack length and width, representing a substantial improvement in measurement reliability. UGV-based systems without manipulators can detect cracks with reasonable consistency but lack adaptive sensor positioning, which reduces accuracy in crack-dimension quantification [29]. By integrating a 2-DoF manipulator with stereo vision and image stitching, the present method enables localized scanning and accurate 3D reconstruction, thereby overcoming this limitation. Moreover, UAV-based platforms are often constrained in GPS-denied or confined indoor environments, while many UGV systems rely on semi-autonomous modes such as wall following [29]. In contrast, the proposed approach combines SLAM-based autonomous navigation with manipulator-assisted inspection, ensuring robust operation in complex indoor settings. Collectively, these advantages demonstrate the novelty of the proposed system, which uniquely achieves high-precision quantification, robustness in indoor conditions, and a high degree of autonomy.

## 6. Conclusions

In this study, a mobile ground vehicle equipped with a 2-degree-of-freedom manipulator was developed. Stereo vision sensors were mounted on both the front of the robot body and the end effector of the manipulator. The front-mounted sensor captured point cloud data for grid-based SLAM, enabling autonomous navigation and real-time mapping, while the manipulator-mounted sensor was utilized for crack detection and dimension measurement. The key outcomes are summarized as follows:

- The mobile robot successfully performed autonomous navigation and real-time mapping using grid-based SLAM with point cloud data.
- The proposed 2-DoF motorized rotational and linear manipulator, combined with a manual rotation plate, enhanced accessibility and expanded the field of view for crack monitoring.
- For crack detection, YOLOv6-s achieved mAP values of 89.6% for horizontal cracks and 79.6% for vertical cracks.
- For crack segmentation, SFNet (ResNet-18) was selected, yielding IoU values of 71.9% and 71.2% for horizontal and vertical cracks, respectively.
- Three-dimensional crack quantification demonstrated high accuracy, with maximum absolute errors of 7 mm (0.70%) for crack length and 0.15 mm (1.00%) for crack width.

Indoor experimental tests confirmed that the developed system represents a reliable solution for automated crack inspection. In the future, the limitations of grid-based SLAM, particularly discretization artifacts on smooth and continuous surfaces, will be addressed by adopting alternative strategies, either through graph-based SLAM using the same sensors or by incorporating additional modalities such as visual–inertial odometry (VIO), LiDAR–inertial odometry (LIO), or LiDAR odometry and mapping (LOAM) to enhance mapping fidelity in complex environments. Moreover, as this study was validated mainly under controlled indoor settings, future work will extend the framework to outdoor field tests under varying lighting, weather, and surface conditions and will also investigate adaptive cropping strategies based on crack morphology to further enhance segmentation performance and robustness.

## Figures and Tables

**Table 1:** Crack-detection performance with and without synthetic data (SD).

	Detection Metrics			Average Precision Metrics	
	Precision	Recall	F1 Score	mAP@0.5	mAP@0.5:0.95
Without SD	0.74	0.53	0.62	0.59	0.40
With SD	0.98	0.98	0.98	0.99	0.87

**Table 2:** Results of crack segmentation using four different deep learning models.

Model	mIoU	Params (M)
SFNet (ResNet-18) [51]	81.3%	13.8
HrSegNet [20]	79.1%	9.7
PP-MobileSeg [52]	78.4%	2.9
RTFormer [53]	77.8%	19.4

**Table 3:** Experimental setup under different conditions: (a) normal lighting with a smooth surface (Case 1), (b) low lighting with a smooth surface (Case 2), and (c) normal lighting with a concrete surface (Case 3).

		Horizontal Length				Vertical Length			
		−50 cm	0 cm	50 cm	100 cm	−50 cm	0 cm	50 cm	100 cm
Case 1	Absolute error (mm)	0.14	0.11	0.13	0.32	0.45	0.02	0.26	0.56
	Relative error (%)	0.24	0.18	0.22	0.53	0.75	0.03	0.44	0.94
Case 2	Absolute error (mm)	0.28	0.36	0.06	0.22	0.17	0.15	0.20	0.22
	Relative error (%)	0.46	0.60	0.11	0.37	0.28	0.25	0.33	0.36
Case 3	Absolute error (mm)	0.42	0.08	0.42	0.24	0.18	0.13	0.18	0.51
	Relative error (%)	0.70	0.14	0.70	0.41	0.30	0.22	0.30	0.85

**Table 4:** Parameters used in the autonomous navigation of the mobile robot.

Process	Parameter	Value
SLAM	Scan frequency	1
	Minimum distance (m)	1
	Minimum angular rot. (rad)	0.5
	Map grid cell size (cm)	5
	Number of particles	30
	Maximum usable range (m)	80
Obstacle avoidance	Detection distance (cm)	60
Path following	Waypoint reach threshold (m)	1.1

**Table 5:** Absolute and relative errors of reference and estimated crack measurements. The values are presented considering the resolution of the reference measurements.

Case	Estimated Length		Estimated Width	
	Abs. Error (mm)	Rel. Error (%)	Abs. Error (mm)	Rel. Error (%)
Horizontal crack				
Width: 10 mm; Length: 588 mm	1	0.17	0.10	1.00
Vertical crack				
Width: 39.5 mm; Length: 995 mm	7	0.70	0.15	0.38

## Data Availability

The synthesized datasets presented in this study are available on request from the corresponding author.
